# Creating a public space and dialogue on sexuality and rights: a case study from Bangladesh

**DOI:** 10.1186/1478-4505-9-S1-S12

**Published:** 2011-06-16

**Authors:** Sabina Faiz Rashid, Hilary Standing, Mahrukh Mohiuddin, Farah Mahjabeen Ahmed

**Affiliations:** 1 James P.Grant School of Public Health, BRAC University, 66 Mohakhali, Dhaka 1212, Bangladesh; 2 Institute of Development Studies, University of Sussex, UK

## Abstract

This article describes and analyses a research based engagement by a university school of public health in Bangladesh aimed at raising public debate on sexuality and rights and making issues such as discrimination more visible to policy makers and other key stakeholders in a challenging context. The impetus for this work came from participation in an international research programme with a particular interest in bridging international and local understandings of sexual and reproductive rights. The research team worked to create a platform to broaden discussions on sexuality and rights by building on a number of research activities on rural and urban men’s and women’s sexual health concerns, and on changing concepts of sexuality and understandings of sexual rights among specific population groups in Dhaka city, including sexual minorities. Linked to this on-going process of improving the evidence base, there has been a series of learning and capacity building activities over the last four years consisting of training workshops, meetings, conferences and dialogues. These brought together different configurations of stakeholders – members of sexual minorities, academics, service providers, advocacy organisations, media and policy makers. This process contributed to developing more effective advocacy strategies through challenging representations of sexuality and rights in the public domain. Gradually, these efforts brought visibility to hidden or stigmatised sexuality and rights issues through interim outcomes that have created important steps towards changing attitudes and policies. These included creating safe spaces for sexual minorities to meet and strategise, development of learning materials for university students and engagement with legal rights groups on sexual rights. Through this process, it was found to be possible to create a public space and dialogue on sexuality and rights in a conservative and challenging environment like Bangladesh by bringing together a diverse group of stakeholders to successfully challenge representations of sexuality in the public arena. A further challenge for BRAC University has been to assess its role as a teaching and research organisation, and find a balance between the two roles of research and activism in doing work on sexuality issues in a very sensitive political context.

## Introduction

The International Conference on Population and Development (ICPD) in Cairo in 1994 put reproductive rights on the international agenda for the first time. In the following year in Beijing, the Fourth World Conference on Women put sexuality on the human rights map. In 2000, the committee for implementation of the International Covenant on Economic, Social, and Cultural Rights (ICESCR), which was first adopted by the United Nations General Assembly in 1966, included for the first time, non-discrimination on the basic of sexual orientation in a statement on the State’s responsibility with regard to health. More recently, the World Health Organization has produced a working definition of sexuality:

‘Sexuality is a central aspect of being human throughout life and encompasses sex, gender identities and roles, sexual orientation, eroticism, pleasure, intimacy and reproduction. Sexuality is experienced and expressed in thoughts, fantasies, desires, beliefs, attitudes, values, behaviors, practices, roles and relationships. While sexuality can include all of these dimensions, not all of them are always experienced or expressed. Sexuality is influenced by the interaction of biological, psychological, social, economic, political, cultural, ethical, legal, historical, religious and spiritual factors” [1]

International advocacy efforts have therefore played a major role in raising the profile of sexuality, gender and rights to become important global issues. At the same time, governments around the world have become more concerned about the public health implications of different sexual behaviours in their efforts to understand and counter the spread of HIV/AIDS and other sexually transmitted diseases [2]. As a consequence, many governments in South and Southeast Asia have changed their policies, expanding reproductive services, and focusing on sexually transmitted infections (STIs) and HIV/AIDS prevention activities. Activism by non government and advocacy organisations has also led to pressure on governments to pay attention to these issues. While many of these activists would argue that not all the demands of sexuality can be addressed by a focus on health, they acknowledge that the AIDS epidemic has forced open spaces for debate about sexuality to gain greater prominence and has been a useful vehicle for raising awareness of different sexualities and sexual rights., However, this has also led to problematizing of sexuality into a sexual health and HIV/AIDS issue with a focus on certain (implicitly high risk) groups [2,3]. The open space therefore often remains circumscribed, and while the concept of sexual rights has gained momentum in the last decade, there has also been increasing resistance from traditionalist forces, including religious bodies as well as governments [2].

In Bangladesh, a paradoxical situation exists, where historically non-normative genders and sexualities, such as the publicly visible *hijras* (transgender communities) are part of the social fabric, albeit marginalised and subject to discrimination. At the same time, there continues to be a culture of collective denial of the existence of same sex sexualities in the country, a fact perhaps linked to religious sentiments, therefore stifling public debate. In the national space, there is a complete lack of public discourse on homosexuality and an overtly heterosexual discourse of marriage and reproduction, which has not allowed other sexualities any legitimacy status. Since 2000, however, some underground groups of gay men have slowly emerged mainly among middle and upper class metropolitan men, who have quietly begun using public spaces and the internet to meet others [4] but remain closeted, with family members, spouses, friends and neighbours unaware of their sexual orientation. Owing perhaps to the strongly patriarchal relations in Bangladesh, no visible female same sex subculture exists and even in “LGBT” (Lesbian, Gay, Bisexual and Transgender) identified groups most of which are still internet based there are very few lesbian identified members. There is some evidence from the informal meetings, research and workshop activities held as part of this project that suggests same sex female sexual orientation exists in Bangladesh with numbers involved reportedly running to more than a 1000 in Dhaka city alone, but they exist in secrecy. This was found to be the case in an informal meeting on November 15, 2009, with sex workers, transgenders and lesbian women from poorer socio-economic backgrounds, and they shared that a network existed of more than a 1000 women (including students, young girls, sex workers, housewives), but very few were willing to come forward to talk about their sexual identity.

In the wake of the Cairo and Beijing Conferences, the government under the umbrella of HIV/AIDS interventions has allowed a number of non-government organisations to reach out to and work with gay men, referred to as ‘MSM’ (men who have sex with men) populations across Bangladesh, but many of these men remain hidden.

The country retains the infamous British colonial anti-sodomy law known as Section 377 of the Penal Code which criminalizes sexuality against the ‘order of nature’ an ambiguous phrase that can be stretched to penalize sexual practices such as heterosexual anal sex, cunnilingus and fellatio. The punishments for crimes perpetrated under this section include fines and imprisonment of up to 10 years. Although the reality is that individuals are rarely prosecuted, they live in constant fear of harassment, ridicule and public shaming from law enforcement officials and the community [4]. The lack of protection of sexual rights of homosexuals and transgender groups and criminalisation of specific practices by the State results in harassment, silence, shame and fear around any discussions regarding sexuality, pushing many issues underground [4,5].

It is against this background that a research team at the James P Grant School of Public Health (JPGSPH), BRAC University in Dhaka began working in 2007 to create a platform to broaden discussions on sexuality and rights, away from an exclusive focus on HIV/AIDS and sexual health. Despite the progress noted above on rights framings, in general, the public framing of sexuality discussions remains very much embedded within a biomedical HIV/AIDS discourse, and is largely treated as a health sector issue. As Cornwall and Jolly [3] note, sex and sexuality is also treated mainly as a source of risk and vulnerability rather than one of rights and positive wellbeing. Building on its local research knowledge, the team hosted meetings, workshops, forums and public dialogues to open up discussions of sexuality among a wide range of stakeholders using positive and human rights framings and as part of a broader conception of sexual and reproductive health rights that starts from local understandings of rights. It was felt that, as a school of public health, we were well positioned in terms of academic legitimacy to play this kind of role and to begin to challenge the silence and discrimination which characterises this important aspect of human experience.

This article presents and examines the experience of linking research on sexuality and rights to active engagement in advocacy with sexual minorities in a challenging context. The article aims to add to the wider literature on using research to get difficult and controversial issues into the domain of public debate through a case from a developing country which is predominantly Muslim and socially conservative.

## Discussion

The case presented here has a number of different elements which interweave contextual factors, specific research activities, development of informed advocacy, learning events and process reflection. The methodology is therefore presented partly as a chronology of these various elements. The work would not have originated without the impetus provided by the research team’s participation in an international research consortium on sexual and reproductive health and rights which began in 2005 and brought together partners in the UK, Kenya, Ghana and Bangladesh. The focus on rights and on sexual health introduced new concepts and discourses which challenged the team’s public health focused approach but which also meshed well with the team’s strengths in qualitative methodologies. This exposure through workshops, conferences and informal interactions across the consortium, contributed to a new research direction for JPGSPH. Subsequently the research team has undertaken research projects, reviewing secondary sources and using both survey and in-depth qualitative methods to examine rural and urban men’s and women’s sexual health concerns and where they go for information and treatment. Additionally over the past year, exploratory qualitative research has been conducted on changing concepts of sexuality and understandings of sexual rights among specific population groups in Dhaka city, including sexual minorities to get a baseline from which to assess change over time.

Linked to this on-going process of improving the evidence base on issues of sexuality and rights, has been a series of learning and capacity building activities over the last four years to use the research insights generated for more effective advocacy. These events have brought together different configurations of stakeholders including members of sexual minorities as well as academics, service providers, advocacy organisations and policy makers.

A third element has been the on-going process of reflection on our experiences with building a platform and space for discussions on sexuality and rights issues in the country. The initiative on learning policy lessons for sexual and reproductive health and HIV/AIDS research which is the subject of this Special Supplement and which brought together four research consortia grappling with different aspects of getting research into policy and practice, provided an invaluable opportunity to try to systematise our thinking. Preparing and presenting the Bangladesh case study for a meeting on the work of the Consortia in Liverpool in 2009 challenged us to undertake a deliberately reflective approach to this work and what it is trying to achieve. A series of discussions and brainstorming sessions on some of our strategies (both planned and unplanned) took place before and after this meeting. We analysed the meeting notes from the various workshops, checked the evaluations of our training and mapped out some of the ways in which we have developed partnerships and alliances in the country and internationally because of our research and advocacy work. We received feedback on the presentation which allowed us to further reflect on our activities on the ground. The reflection therefore involved a combination of individual reflection by us (as authors of this paper) and collaboratively with discussions between colleagues and some of our international partners.

### Conceptual framework

It is well recognised now that the process of using research to influence policy or implementation for specific ends is complex, context and issue dependent and often opportunistic [6]. In this particular case, the desired outcomes were themselves relatively open beyond the basic commitment to research as a way of creating a more enabling public climate for open discussion of sexuality and rights. This was seen as a necessary pre-requisite for advancing a rights based culture conducive to good public health and a supportive policy and legal framework. In analysing this particular case and particularly in understanding pathways to influence, we have drawn upon or adapted aspects of several different policy process conceptual frameworks. Common to all or most frameworks is an emphasis on power and process as integral to policy influence and on context, institutions and actors as key elements of policy making and influence [7-11]. At a broad level, the Overseas Development Institute’s Research and Policy in Development Programme (RAPID) framework [10] for understanding research-policy links is useful in identifying critical conceptual elements of the process in terms of overlapping spheres of context (politics), evidence (credibility), links (influencing actors) and external influences. Sumner et.al. [11] importantly add “values” to the critical elements of context, institutions and actors. These elements provide the *institutional* framing of research influencing work.

In analysing the activities and processes through which researchers seek to have impact or influence, Sumner et.al. [11] draw attention to the overlapping ingredients of research influence in the form of sticky messages or rallying ideas, “knit-working” or coalition building and championing of ideas, and strategic opportunism or finding/creating windows of opportunity. These capture the often intuitive, untidy and responsive ways in which this kind of work proceeds and address the operational dimensions of influencing. Finally, the concept of different policy spaces [12-14] opens up the question of “what” and “who” in using research for influencing. The spaces to be influenced may be closed, formal spaces within bureaucracies; invited spaces such as public or expert consultations; political/electoral spaces; “claimed” or popular spaces for advocacy and conceptual spaces which introduce new ideas into public discourse. Spaces have their own dynamics and may be more or less visible.

These three overlapping conceptual arenas – contextual and institutional framing, operational approaches and influencing spaces – provide a useful way of reflecting on and organising our experience.

### Context, institutions and actors

The international and national contexts for sexual health and rights have been described briefly in the background section. The specific impetus to push this agenda forward came from an initial international meeting on this topic at the Institute of Development Studies in the UK in 2005. The first author, inspired by this and after returning to Dhaka, took the first step of locating interested persons and holding a small local workshop in January 2007. In the current climate in Bangladesh, sexual rights of minorities are severely undermined by the lack of a supportive legal and policy environment which constrains the level of engagement and impact possible. There is ongoing discrimination and violence against sexual minorities (e.g. gay men, transgender) especially by the police who, instead of protecting them, routinely subject them to violence, extortion and sexual abuse. In the case of transgender groups, there are issues such as the fact that they cannot have passports as they do not fall neatly into binary categories of male or female. There were thus important values dimensions behind this impetus. The research team from the start used or created links with advocacy communities working from a human rights perspective.

For the initial activity, the team worked quietly relying on informal networks to organise the workshop, but as word spread, the interest grew to include some 30 participants who were individual researchers, sexual minority groups, activists and researchers working in concerned organizations. At the workshop, they presented papers and brainstormed on how one could move the agenda forward. From this meeting there was a realization that despite the silence, there was much enthusiasm and support for moving sexuality discussions out into the public arena. This was a crucial turning point as many of the participants at this initial meeting identified colleagues, friends, NGOs, and other stakeholders in Bangladesh and abroad working on and promoting sexuality and rights. The team perceived that there was an urgent need for a broader approach and conversation about sexuality and rights in the larger community.

This led to the School of Public Health hosting an international conference on Gender and Sexuality in July 2007 with prominent speakers and activists from Turkey, India, Pakistan, USA, United Kingdom, Kenya and Bangladesh who contributed papers, with over 150 participants attending. The workshop covered a range of topics such as society's prejudices towards minority groups, and issues faced by LGBT members in living in a heterosexually dominated society. As the conference was being held at the BRAC Centre and hosted by the University, this gave the subject matter legitimacy and credibility. We therefore decided to invite a diverse group of policy actors and practitioners (women’s groups, activists, researchers, academics, media professionals, students, and health providers, NGOs and gay, lesbian and transgender groups).

Dialogue about sexual and reproductive health and rights among scholars, policymakers, researchers, activists and practitioners tends to be insufficient, with discussions around sexuality and rights neglected. In the NGO sector, development interventions that address reproductive and sexual rights are health-focused with the rights approach largely missing. Due to the lack of public discussions on sexuality, we realised early on that we needed to test out the scenario and engage with a broader base initially to expose them to different ideas, constructs of sexuality and rights and to challenge their traditional understandings, thus changing the way sexuality and rights was viewed, discussed, practised and implemented.

Soon after the International conference in 2007, the School was approached by the International Women’s Health Coalition (IWHC), interested to fund further activities on sexuality and rights in Bangladesh. Two training workshops and a follow up workshop were held with this funding. Three core groups were targeted. One group was academics from outside Dhaka to encourage them to develop sexuality dissemination activities and provide a space for discussions among academics and students outside of Dhaka, who are often overlooked in metropolitan debates. Another group was journalists and advertising agencies to encourage writings on sexuality and discuss representations (and the absence of) and constructions of sexuality in the media and in advertisements. Currently in Bangladesh, there is nothing written on the diversity of sexualities, or often these issues are biased or misrepresented. The third group was the LGBT community who remain mainly underground, with only some visibility of MSM and transgender groups who work under the umbrella of HIV/AIDS organizations. The idea was to provide non-conforming sexual groups the capacity and space to argue for advocacy, training and build their own agenda to fight for their basic rights.

It is important to point out that within these groups, tensions exist on ‘representations’ and language to discuss LBGT identity in the country. For example, there is disagreement regarding the popular terminology of MSM (men who have sex with men) to refer to gay men, amongst NGOs who work on HIV/AIDS intervention. This term is strongly disliked by primarily well off and educated self identified ‘gay’ men who view the term as degrading, whereas among the less well off MSM population, the term is not seen as problematic at all, but speaks to their identity and the reality of their lives. Similarly among the ‘lesbian’ groups, there is no clear affinity among the few self identified middle class women (be it younger or older), and initial meetings found that one group of women prefer the term ‘lesbian’ and another group preferred the term ‘women who love women.’ It is not clear as to level of dialogue or support between middle income earning and poorer ‘lesbian’ women networks, where access to technology such as a recent internet website set up for and by ‘lesbians,’ is a luxury few of the poorer classes of lesbian women can afford, access or even understand given their education. Middle class ‘lesbian’ women are fighting for legal recognition and acceptance, whereas for poorer ‘lesbian’ women, their concerns revolve around basic survival (having access to food, shelter and livelihoods). If one brings the transgender community into this picture, their needs and priorities also differ as they are a very visible community, and live out their lives and identity (sexuality) in public spaces and yet are constantly subject to violence and discrimination.

### Realities of building a platform

Given that historically there had not been a conference like this in Dhaka city, we were rather naïve and did not even think of the possibilities of a backlash from the audience or the media. In fact, there was widespread positive interest and coverage of the conference and of sexual minorities in some of the leading English and Bengali newspapers in the country. This was new, given that much of the representation in these newspapers is either HIV/AIDS related when speaking of gay men or focused on *hijra* communities with negative stereotypes prevailing.

Policy process analysis emphasises the importance of the credibility, relevance and usefulness of research evidence in terms of its influencing value [8,15]. The School had already built credibility through its research on neglected areas of sexual and reproductive health in rural and urban Bangladesh. In 2008, a local group of academics, advocates and people from different agencies was convened for a brainstorming session on research priorities in sexuality and rights. This resulted in the development of a research project to understand local constructions of sexuality and rights among mainstream urban students in public universities, gays, lesbians and transgender community, female garment factory workers and staff of NGOs and other agencies working in this area. These groups were chosen self-consciously as likely to represent changing attitudes and behaviours. The aim of the research was to contribute to filling knowledge gaps in this area, and to share findings in future workshops, training and advocacy efforts. This led to the creation of a small active network of core stakeholders working in or interested in taking forward sexuality and rights issues including mental health professionals, researchers, academics, activists, LGBT groups, students, journalists, lawyers and health educators and activists.

A number of factors shaped the manner in which we engaged in and planned our activities after the success of the International conference. Timing and luck played a part (the role of ‘serendipity’); for instance being approached by interested funders at the right time. It was also a case of identifying and seizing opportunities – what Sumner et al [11] refer to as ‘strategic opportunism.’ For instance, from the international meeting we developed a resource pack which was a compilation of all the presentations from the International conference in 2007. This was distributed to more than 150 stakeholders all over Bangladesh. We then received more than 50 emails and 60 to 70 phone calls from participants, academics, journalists and interested NGO practitioners from within and outside of Dhaka requesting more materials and resources. Academics outside of Dhaka specifically requested more training to develop their own modules on sexuality. Clinical psychologists and doctors expressed interest in receiving training to be sensitized to sexual minorities.

Some of the advocacy strategies undertaken by the Centre were deliberate, and following much reflection, and at other times they were about ‘right timing’ and seizing opportunities as they arose, taking intuitive decisions, networking through informal contacts and personal relationships, exploring innovative ways to push the boundaries on sexuality. Sometimes events just unfolded (e.g. positive media coverage of our first ever International Conference in 2007, being noticed by and receiving funding from an international organization for advocacy work) which paved the way for new and further strategies, such as providing training to targeted groups, building a list-serve of members, and conducting a new research study on sexuality and rights to understand the local context and situation. Methodologies of engagement were not always planned ahead of time but designed and implemented as needed to address emerging concerns and demands as the coalition of interested stakeholders grew. Throughout this period, we initiated small meetings and forums and hosted some series of films on sexual diversity, inviting students and partners to attend. We also hosted a meeting to discuss the implications of the Bangladesh penal code 377, and more recently a meeting on a South Asian network working on human rights for LGBT communities across South Asia.

### Influencing spaces

As was noted earlier, this work was originated with a relatively open view of potential outcomes. However, there was a clear understanding that the important space to be influenced was the conceptual space. Given a combination of the resources we had available and our positioning as an academic institution, the principal focus of the work was to use research and informed debate to challenge representations of sexuality and rights in the public domain. As the work progressed through activities to build partnerships and alliances, other spaces have become engaged. The increasingly visible participation of LGBT groups and strengthening of advocacy in this arena has begun to open up claimed or popular spaces where such groups feel able to contribute their own voices. For example, in some of our training workshops in 2009, LGBT groups were invited as participants and encouraged to share experiences and challenge existing hetero-normative frameworks with a diverse group of participants. This also allowed for LGBT members to reach out to each other and start the dialogue with one another. It is hoped that other policy spaces can be opened up through this unfolding process, for instance in relation to law and discriminatory practices.

### Assessing and measuring influence – the role of interim outcomes

This process has brought visibility to sexuality and rights issues which were otherwise hidden, through the process of ‘knitworking’ – forging strategic networks and alliances [13] – with small successes which are often difficult to measure. Interim outcomes are steps along the way, smaller changes that need to happen before final outcomes can be reached [16]. According to empirical studies this kind of impact is more common than direct impact [11,17]. Given this context, how does one develop appropriate methodologies or strategies to measure this kind of impact especially on an issue which is socially taboo and considered invisible in the larger environment?

After our International Conference in 2007 and workshops in 2009, academics who participated from two universities outside of Dhaka, pledged to offer short modules on sexuality and rights in their respective departments in 2010. They were also keen to host their own conference on sexuality and rights regionally in 2010. The research on local constructions of sexuality has led to sensitizing of groups through focus group discussions which created a space for frank and honest discussions amongst respondents themselves. Through the researchers’ interaction with students in Dhaka’s public universities, there has also been significant interest generated among the students in terms of wanting to know more about sexual diversity and its relationship to rights, and linkage to health and wellbeing. One of the other main successes of the research approach was gaining credibility and trust among sexual minority groups. A methodological research strategy was the active involvement of members from these groups to gain access to ‘underground’ members of the communities who were more reluctant to participate directly. This ensured better confidentiality and anonymity of the participants and led to further confidence on the research and better sharing of the experiences of the participants. Gay and lesbian rights groups who had primarily been very reserved and cautious in terms of allowing access to people outside of their communities have gained confidence through the research activities undertaken by the Centre. Two of the authors of this study were invited to a two-day workshop on Sexual Diversity and Coalition Building organized by a gay rights activists group in March 2009. This was an unpublicized meeting with important representatives from the LGBT communities aiming to build a network and come up with strategies for gaining positive visibility in society. The LGBT communities are setting up a Coalition and have approached the School to provide venue support for their trainings and access to resource materials for training of their members. Given the level of trust and close relationship with many members of the Coalition, staff members were invited to the first ever historic inauguration of a registered office for the Coalition of LGBT in Bangladesh in August 2009. Donors have recently provided support to fund the establishment of an office for the Coalition of LGBT in Dhaka.

There were some open declarations of sexual preferences at the international conference in 2007 and in the training workshops in 2009. In the international conference, two gay men for the first time spoke openly of their sexual orientation in this visible space. There was also a speaker from India who had established a lesbian advocacy group and shared her personal life story and advocacy experiences. In one of the training workshops in 2009, there was for the first time representation from both marginalised and mainstream communities - self identified gay men from middle class backgrounds, lesbians, transgenders, academics and researchers, who worked together. In this workshop a number of participants shared their experiences of how their attitudes towards the transgender communities had changed: *‘before when I would see a hijra* (*transgender*), *I would walk over to the other side of the road to avoid them*, *but now I will go up and say hello and give them a smile. They are also human beings like us.*’ These public declarations and discussions were received with applause from many members in the audience, much to our surprise. A more striking indicator of success was the presence of two members of the ‘lesbian’ community two years later at the Gender training workshop in May 2009, with one of the women identifying her sexual orientation in a very public forum. It is important to point out that the not all of the women identify with the term lesbian; and the woman and her friend who attended the workshop instead identified themselves as ‘shomopremi nari’ (women who love women – a phrase which intentionally does not have sexual connotations). Her foundation set up a support network for other sexually marginalised women. The presence of the two women was in stark contrast to efforts in 2007, where it was impossible to get any representation openly from this community in Bangladesh at our International conference.

As part of monitoring the impact of the workshops on participants, pre and post evaluation questionnaires were handed out among all participants to map existing general knowledge and expectations, to understand level of awareness and future proposed activities after the workshops. There was a follow up workshop inviting participants back to discuss and share experiences of the workshops, and any changes from the training in their personal or professional lives. Moreover, we set up an awards system to actively encourage journalists to write news stories on sexuality and rights in local and regional newspapers, by giving a small monetary award for any news item/story published. Initial reports indicate that at least five regional journalists had written pieces on Sexuality and Rights, which were published in local newspapers, and one journalist had developed a radio story to be broadcast.

In the international arena, the Centre for Gender, Sexuality and HIV/AIDS at JPGSPH was invited by the Coalition for Bodily and Sexual Rights (CSBR), whose headquarters are in Turkey, to become a member of their International Advocacy group. This comprises members and networks from all over the world. International Women’s Health Coalition has indicated interest in continuing to fund the Centre’s advocacy activities and new interest has been generated from other donors, which allows for building capacity and continuity of advocacy efforts in the future. The availability of modest funds has provided the Centre with autonomy and space to reach out to its networks and continue to establish new connections with many diverse groups/actors and stakeholders in the country.

### Challenges

Gilson and McIntyre [17] suggest that there needs to be more investment in understanding the actual processes of policy development itself, at all organizational levels. For instance, research for advocacy is often ‘time bound’ as institutional actors change positions. In terms of research itself, one influence over its potential impact is its legitimacy amongst end users [17]. The reluctance of the politicians to favour or endorse rights arguments on this issue has been noticed in most countries struggling to create space for all sexual identities. Debates held in India after Section 377 was repealed by the High Court indicated that even progressive leaning political personalities hesitated to clearly state their positions and reaction to this change. One of the realisations from the workshops held at the Centre for Gender, Sexuality and HIV/AIDS in Bangladesh has been that politically influential people who are closer to power may need to be sensitized further through different approaches for significant attitudinal changes to take place in the society at large.

Given the present political climate, and the nature of the subject matter and lack of an enabling environment in Bangladesh, it is very difficult to predict the level of impact our research/advocacy will have on any larger change in government policy in the immediate term. Very few governments will push forward policy changes which may be viewed unfavourably in the wider society. However, we found that it is possible to create a public space and dialogue on sexuality and rights in a conservative and challenging environment like Bangladesh by bringing together a diverse group of stakeholders – LGBT groups, research, academics, NGO professionals, health providers, journalists and policymakers – to successfully challenge representations of sexuality in the public arena.

Any long term initiative focused on policy impact also needs financial sustainability. Many activists/human rights organizations have limited resources and capacity in areas of sexuality research and advocacy. This is a new area and there is little in terms of support in the wider arena and multiple agendas among diverse stakeholders (e.g. donors and NGOs), limited resources and the absence of a larger movement on sexuality among mainstream advocacy and activist organizations relegate such issues to the background. Although the realization of the importance of a rights-based approach exists among the NGO community in Bangladesh, it has not been substantially translated into practice. One of the major challenges remains fighting a commonly held attitude in the government and among many NGOs that homosexuality is ‘illegal’, ‘culturally and religiously unacceptable’ and an ‘imported idea from the West’; while some are of the opinion that discussions of sexuality and rights are a luxury against the backdrop of poverty and other priorities for the country.

Against this backdrop, we have also noted the problem of how to track more subtle changes, such as changes in behaviours and attitudes among stakeholders, policymakers given the silence surrounding many of these issues. This continues to remain a challenge but we have argued that tracking interim outcomes provides an important way of capturing the processes which may precipitate more fundamental change. A recent report from Organizational Research Services (ORS) offering guidance to advocacy organisations on creating desired changes points that: “While the focus of advocacy work is often on policy wins and improved conditions for populations and the environment, much of the progress occurs in the landscape along the way” [18].

### Small steps forward

According to this paper, final outcomes require prior structural changes in institutions, systems, beliefs and commitments (ibid). The process described in this paper contributed to effecting change in three of the structural areas identified by the ORS report as critical to final outcomes. The first was widening and strengthening the base of support for advocacy on sexuality and rights through bringing together key actors who were in a position to influence the wider climate, such as the media. The second was strengthening alliances, through providing platforms and spaces for allies to come together. This was done by assessing strategic opportunities and building sustained alliances and partnerships with media, activists, academics and sexual minority groups. The third was through contributing to shifts in social norms around sexuality through creating a more open climate of discussion and dialogue in a wider range of arenas.

Coffman [19] suggests that these kinds of ‘interim outcomes’ should be given the respect they deserve, as they may be as important as policy change itself. We should not assign ‘second class status’ to such outcomes She argues it is important to assess advocacy for more than just its impact on policy, and pay attention to the core components of such efforts, including capacity building, network formation, relationship building, communications, and leadership development which can sustain influence in the larger policy processes. Capturing and reflecting on interim outcomes is an important step in thinking through the strategies groups might need to consider when working on highly politicised and difficult issues in contexts such as this.

## Conclusion

It was found to be possible to create a public space and dialogue on sexuality and rights in a conservative and challenging environment like Bangladesh by bringing together a diverse group of stakeholders to successfully challenge representations of sexuality in the public arena. This work has also led to reflection on the role of an academic institution in advocacy for “policy change.” BRAC University as a teaching and research organisation is doing work on sexuality issues in a very sensitive political context. Some questions remain: what is the role of the School, an academic institution in tracking, monitoring and continuing to push for changes at the national level? What are the responsibilities of the other actors, such as legal institutions, civil society members and so on? While that political context must be addressed and actively engaged with to promote change, academic institutions have to balance their roles as researchers and their responsibilities to citizenship. We conclude that there was an institutional responsibility to use the legitimacy built up by the JPGSH to support and enable advocacy through active engagement in research and coalition building.

## List of abbreviations used

CSBR: Coalition for Bodily and Sexual Rights; ICPD: International Conference on Population and Development; ICESCR: International Covenant on Economic, Social, and Cultural Rights; IWHC: International Women’s Health Coalition; JPGSPH: James P Grant School of Public Health; LGBT: Lesbian, Gay, Bisexual and Transgender; MSM: Men who have sex with men; RAPID: Research and Policy in Development Programme; STI: Sexually Transmitted Infection

## Competing interests

This article critically reflects on a research project in which the authors have been involved.

## Authors' contributions

Sabina Faiz Rashid was the principal investigator for the project. She worked on the conception and design of the study; analysis and interpretation of the data; drafting the first version of the paper and making substantial intellectual inputs and revisions to subsequent drafts. Hilary Standing provided support to the conception and design of the study; analysis and interpretation of the data. She worked on the second and subsequent drafts of the paper and made critical intellectual inputs to the conceptual framework. Mahrukh Mohiuddin was a member of the research team for the Sexuality and Rights project. She worked closely on the conception of the study and contributed to the conclusions section of the paper. Farah Mahjabeen Ahmed was a member of the team for the Sexuality and Rights project. She contributed to the conclusions section of the paper.

## Authors' information

Sabina Faiz Rashid, PhD, is Associate Professor at the James P Grant School of Public Health, BRAC University, Bangladesh

Hilary Standing, PhD is a Professorial Fellow of the Institute of Development Studies, University of Sussex UK and Visiting Professor at the James P Grant School of Public Health, BRAC University, Bangladesh

Mahrukh Mohiuddin, MA, was Senior Research Associate/Lecturer at the James P Grant School of Public Health, BRAC University, Bangladesh from July 2007 to August 2009

Farah Mahjabeen Ahmed, MS, is Senior Lecturer at the James P Grant School of Public Health, BRAC University, Bangladesh
